# The Effects of YAP and Its Related Mechanisms in Central Nervous System Diseases

**DOI:** 10.3389/fnins.2020.00595

**Published:** 2020-06-26

**Authors:** Jiayan Jin, Xiaoxuan Zhao, Huifang Fu, Yuan Gao

**Affiliations:** ^1^Department of Shanghai Key Laboratory of Forensic Medicine, Shanghai Forensic Service Platform, Academy of Forensic Science, Shanghai, China; ^2^Department of Forensic Science, School of Basic Medical Science, Wenzhou Medical University, Wenzhou, China; ^3^Department of Pathology, Traditional Chinese Medicine Hospital of Jiangning District, Nanjing, China; ^4^Forensic Center, Wenzhou Medical University, Wenzhou, China; ^5^School of the 2nd Clinical Medical Sciences, Wenzhou Medical University, Wenzhou, China

**Keywords:** central nervous system diseases, Hippo signaling pathway, YAP, effect, mechanism

## Abstract

Yes-associated protein (YAP) is a key effector downstream of the Hippo signaling pathway and plays an important role in the development of the physiology and pathology of the central nervous system (CNS), especially regulating cell proliferation, differentiation, migration, and apoptosis. However, the roles and underlying mechanisms of YAP in CNS diseases are still puzzling. Here, this review will systematically and comprehensively summarize the biological feature, pathological role, and underlying mechanisms of YAP in normal and pathologic CNS, which aims to provide insights into the potential molecular targets and new therapeutic strategies for CNS diseases.

## Introduction

**FIGURE 1:**
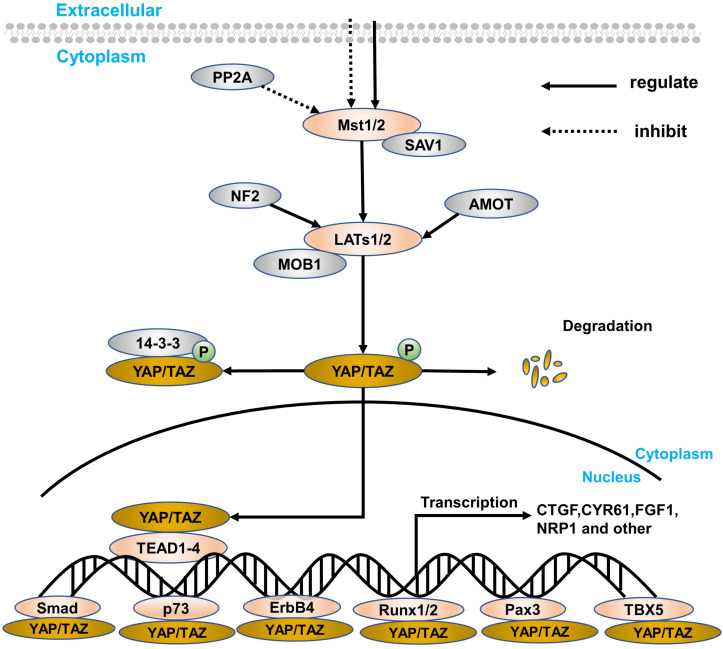
A simple overview of the Hippo signaling pathway and YAP. The core components of the Hippo signaling pathway are Mst1/2, LATs1/2, YAP/TAZ, and target genes, which are closely connected, especially YAP. The retention method for YAP is in the nucleus and cytoplasm. In the cytoplasm, YAP/TAZ can blind to some specific proteins (such as the 14-3-3 protein) through direct LATs1/2 phosphorylation and indirect Mst1/2 activation. At the same time, SAV1 and MOB1 are defined as regulatory proteins of Mst1/2 and LATs1/2, respectively. Besides, PP2A can inhibit the Mst1/2–SAV1 complex, while both NF2 and AMOT can regulate the LATs1/2–MOB1 complex. In the nucleus transferred from the cytoplasm, YAP can express a series of target genes through interacting with transcription factors, like TEAD family, Smad, p73, ErbB4, Runx1/2, Pax, and TBX5. Finally, when suffering from certain physiological and pathological damage, YAP will degrade.

The Hippo signaling pathway was first discovered in the Drosophila wts gene mutation, causing the growth phenotype of the eyes and wings (Justice et al., 1995). However, the components of the signaling pathway are highly conserved from *Drosophila* to mammals (Zhao et al., 2010a), including mammalian STE20-like protein kinase 1/2 (Mst1/2), Salvador family WW domain-containing protein 1 (SAV1), large tumor suppressor substance 1/2 (LATs1/2), MOB kinase activator (MOB1), Yes-associated protein (YAP) and its paralog, and TAZ and TEA domain-containing sequence-specific transcription factors (TEAD1–4; Chan et al., 2005; Lei et al., 2008; Wang et al., 2019). The Hippo signaling pathway is relatively highly conserved in mammals and is mainly composed of a series of core kinases, including MST1/2, SAV1, LATs1/2, and MOB1A/B. When receiving upstream signal stimulation, phosphorylation-activated MST1/2 in turn leads to phosphorylation activation of other core kinases, SAV1, LATs1/2, and MOB1. The interaction between MST1/2 and SAV1 enhances the activity of MST1/2 kinase, which in turn promotes the phosphorylation and activation of LATs1/2. The activation of the Hippo signaling pathway then causes YAP/TAZ phosphorylation to remain in the cytoplasm, eventually leading to its chelation and proteasome-mediated degradation. Conversely, when the Hippo signaling pathway is inactivated, dephosphorylated YAP/TAZ enters the nucleus from the cytoplasm, promoting its downstream gene expression that mediates cell proliferation and migration. Because YAP lacks a DNA-binding domain, YAP needs to combine with other transcription factors to form a transcriptional regulatory complex, such as TEAD, RUNT-related transcription factor 1/2 (RUNX1/2), and T-box transcription factor 5 (TBX5), especially TEAD (Zhao et al., 2007, 2010a; Figure 1).

Yes-associated protein is the main downstream effector of the Hippo signaling pathway in mammals. As a transcriptional activator, YAP has no DNA-binding domain and cannot directly bind to DNA. When the signaling pathway is inhibited, it is necessary to interact with some transcription factors to mediate transcription factors of multiple genes (Mo et al., 2014), like CTGF, CYP61, NRP1, and FGF1. However, LATs1/2 phosphorylates YAP, and proteins in the cytoplasm, like 14-3-3 protein and AMOT, are then ubiquitinated and degraded (Figure 1). What’s more amazing is that the position of YAP can regulate the volume of the organ and the regeneration of tissues to maintain cell proliferation and apoptosis and participate in the inhibition of cell contact (Zhao et al., 2007, 2010a). Thus, research regarding the Hippo signaling pathway has been paid more and more attention, and YAP is considered to be the most important downstream effector in the current research (Vassilev et al., 2001; Zhao et al., 2008b, 2010a).

## Role and Mechanism of YAP in the Normal Central Nervous System

As we all know, YAP can regulate the volume of organ and regeneration of injured tissues, maintain the balance of cell proliferation and apoptosis via the downstream gene expression degree, and participate in cell contact inhibition regulation. In this section, we set forth that YAP keeps a balance between apoptosis and proliferation in neural progenitor cell (NPCs) to reach a steady state, responds to neuroinflammation, ensures the smooth signal transmission via downstream pathway or genes, and interacts with endothelial cells with different approaches during angiogenesis (Table 1).

**TABLE 1:** Summary of the role of YAP in normal cells of CNS.

Normal cell	Mechanism	Influence	References
NPC	• Accelerate proliferative division of self-expanding cortical RGCs • Reduce the differentiation of IPCs • The threshold for activating apoptosis is very low • Sensitive to DNA damage	Induce apoptosis and avoid excessive proliferation	(McKinnon, 2017; Lavado et al., 2018)
BV-2 neuroglia cells	• Upregulate Sirt3 and inhibit the JNK pathway • Inhibit apoptosis and promote proliferation to replenish damaged brain tissue • Restore caspase 9 and caspase 12 to normal levels	Respond to neuroinflammation with TNFα treatment	(Biernacki et al., 2018; Gandarillas et al., 2018; Sorrentino et al., 2018; Yang et al., 2019)
Purkinje cells	• Active mTORC1/p70s6k and mTORC2/Akt • Promote normal cerebellum morphology and motor coordination function	Ensure smooth signal transmission with mTOR and p70s6k	(Petritsch et al., 2000; Urbanska et al., 2012; Liu et al., 2017)
Endothelial cells	• YAP/TAZ activity is controlled by VEGF to trigger angiogenesis • Regulate the Rho family GTPase CDC42 activity in the cytoplasm • Interact with STAT3 and Ang-2 • Regulate non-endothelial cells, like pericytes	Finish angiogenesis to form a vascular network	(Justice et al., 1995; Saucedo and Edgar, 2007; Zhao et al., 2010b)

### YAP/TAZ-Activation-Induced Apoptosis in NPCs

NPCs are a kind of precursor cells differentiated from neural stem cells (NSCs) and specifically differentiated into neurons (Lee et al., 2016). Their proliferation, differentiation, and migration are closely related to embryonic development and the number and precise distribution of neurons after birth. The normal size, composition, and function of the nervous system depend on the precise self-renewal and specific differentiation of NPCs. Studying the mechanism of neural precursor cell differentiation and proliferation can also provide a new scientific basis for neurodegenerative diseases and nerve damage. Recent studies have found that the Hippo/YAP signaling pathway plays an important role in the proliferation, differentiation, and apoptosis of NPCs (Lee et al., 2016; Lavado et al., 2018).

**FIGURE 2:**
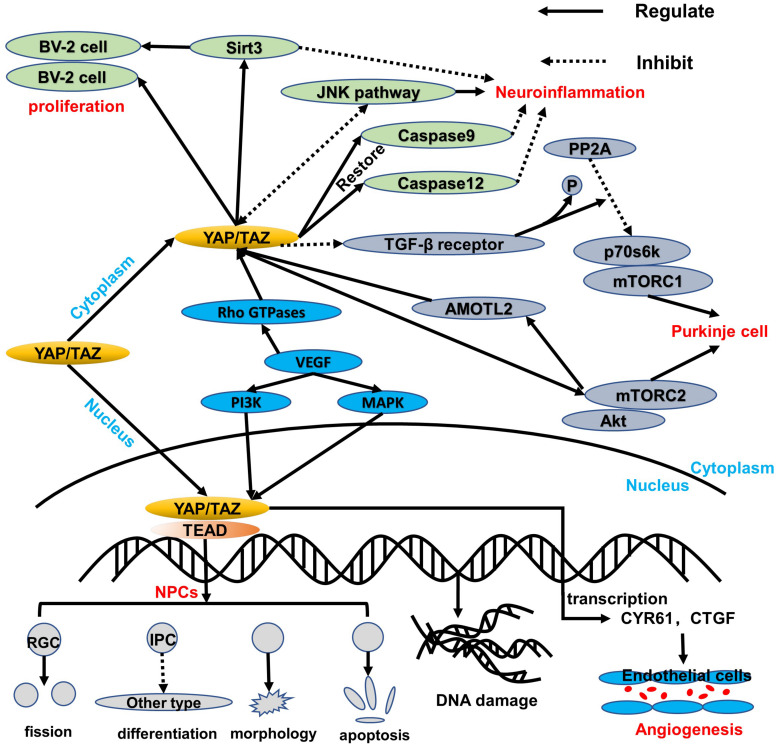
An overview of the regulation of YAP/TAZ in healthy tissue. YAP/TAZ regulates the healthy cells of the central nervous system. In NPCs, YAP/TAZ shifts to the nucleus to interact with the TEAD family, thereby leading to a balance of proliferation and apoptosis via blocking IPC differentiation, damaging morphology, accelerating the RGC fission, and inducing DNA replication stress and damage. Additionally, YAP/TAZ in the cytoplasm can upregulate Sirt3 and inhibit the JNK pathway, which both are beneficial to protect BV-2 cell proliferation and respond to neuroinflammation. Moreover, caspase 9 and caspase 12 can be restored to reduce mitochondrial damage and endoplasmic reticulum stress. Like the BV-2 cells in the cytoplasm, the p70s6k-and-mTORC1 complex is regulated via downregulation of PP2A and TGF-β receptor, while the Akt-and-mTORC2 complex can interact with YAP/TAZ through AMOTL2. More significantly, VEGF induces YAP/TAZ via Rho GTPases and subsequently triggers YAP/TAZ target genes to protect the mitosis and survival of endothelial cells during angiogenesis through PI3K and MAPK regulation.

In the developing neural tube of vertebrates (chicken embryos), the ependymal NPCs express YAP. Overexpression of YAP or transcription-activated TEAD in the neural tube can cause a decrease in neuronal differentiation and a significant increase in the number of NPCs. YAP deficiency causes cell death and promotes early neuronal differentiation. These effects are mainly related to the upregulation of cyclin D1 expression induced by YAP/TEADs. Moreover, conditional knockout of LATs1/2 results in a decrease in phosphorylated YAP and an increase in the level of YAP/TAZ, which triggers the shift of most YAP/TAZ proteins from the cytoplasm to the nucleus. The strong nuclear localization of YAP/TAZ enhances global transcription activity and upregulates many genes, thereby boosting the capacity of biosynthesis and proliferation (Lavado et al., 2018), which is consistent with the known Hippo kinase cascade. Nevertheless, in NPCs, LATs1/2 deletion accelerates proliferative division of self-expanding cortical radial glial cells (RGCs) (Kriegstein and Alvarez-Buylla, 2009) and reduces the differentiation of intermediate progenitor cells (IPCs) (Lavado et al., 2018), which are sufficient to induce apoptosis dependent on TEAD-guided transcription and ultimately exacerbate DNA replication pressure and DNA damage and worsen brain morphology (Figure 2). More interestingly, the mode of action of YAP is distinct from the normal context of the upregulation of antiapoptotic genes, probably because NPCs are particularly sensitive to DNA damage and their threshold for activating apoptosis is very low (McKinnon, 2017). Consequently, the YAP signaling pathway plays an important role in cell proliferation and differentiation. Its overexpression or overactivation can promote both cell proliferation and cell apoptosis, which helps maintain the homeostasis.

### YAP Responds to Neuroinflammation in BV-2 Glial Cells Related to Sirt3 and JNK Pathways

Under the condition of TNFα-induced neuroinflammation, the viability of BV-2 cells decreased while the apoptosis index increased. At the same time, YAP expression is significantly reduced, which indicates that YAP may aggravate BV-2 cell death in neuroinflammation (Yang et al., 2019; Figure 2). Studies have found that overexpression of YAP induced by adenovirus transfection can reverse the viability of BV-2 cells, thereby reducing the apoptosis index of TNFα-treated BV-2 cells. The molecular mechanism study found that YAP overexpression is closely related to Sirt3 upregulation (Cheignon et al., 2018). Sirt3 has been validated as the biomarker of the viability of BV-2 neuroglia cells (Cui et al., 2018) and is required for YAP-induced BV-2 cell protection (Li et al., 2018; Sorrentino et al., 2018; Yang et al., 2019). Increased Sirt3 expression can reduce ER stress response, reduce mitochondrial damage, and block the JNK pro-apoptotic pathway, and the knockout of Sirt3 eliminates the protective effect of YAP overexpression on TNFα-treated BV-2 cells, indicating that YAP overexpression can protect BV-2 cells from TNFα-mediated cells. In addition to Sirt3, the JNK pathway, which is found as the classical downstream pathway of neuroinflammation response (Yang et al., 2019), frequently triggers oxidative stress, endoplasmic reticulum (ER) stress, irradiation, infection, and interaction of the pro-apoptosis genes. Thus, Sirt3 upregulation and JNK pathway inhibition via YAP activation protect BV-2 cells from excessive apoptosis and relieve severe inflammation symptoms in the neuroinflammation microenvironment. Interestingly, reactivation of the JNK pathway activity significantly attenuates the beneficial effects exerted by YAP (Yang et al., 2019), whereas high YAP expression inhibits JNK pathway activity, which may form dynamic circulation stability *in vivo*. Apart from those mentioned above, YAP can also inhibit apoptosis and promote proliferation to replenish damaged brain tissue in BV-2 cells of the neuroinflammation microenvironment (Biernacki et al., 2018; Figure 2). Meanwhile, caspase 9 and caspase 12, respectively restored to normal levels via the upregulation of the YAP level, which can attenuate the alternations of mitochondrial damage and ER stress (Gandarillas et al., 2018; Figure 2). Consequently, YAP of BV-2 cells can exert corresponding defenses to cope with the neuroinflammation microenvironment in physiological conditions.

### YAP Ensures Smooth Signal Transmission via Protecting Purkinje Cells Associated With mTOR and p70s6k

Purkinje cells are an important part of the signal transmission (Fang et al., 2018), and dendritic dysfunction can cause the related signal transmission to be blocked. The current study found that the YAP signaling pathway can maintain the normal structure of the Purkinje cell dendrites, thereby promoting normal cerebellum morphology and motor coordination function (Liu et al., 2017). YAP participates in the regulation of transforming growth factor-β (TGF-β)/Smad signaling (Ferrigno et al., 2002), mainly interacts with Smad7, and inhibits TGF-β receptor activity. Accordingly, the protein phosphatase 2A (PP2A) is regulated via TGF-β dephosphorylation and downregulation of p70s6k (Petritsch et al., 2000), which can also be phosphorylated by mTORC1 (Figure 2). Thus, the complex of mTORC1 and p70s6k, which is defined as one of the significant regulatory regulators of dendritic arbor development (Moser et al., 1997), is required for dendritic arbor development (Urbanska et al., 2012), including the width and number of dendritic branching point (Rojek et al., 2019). In addition to the above, the downstream effectors of mTORC2, which are essential to the growth of dendritic trees, are related to Akt. Even more surprising is that mTORC2 kinase phosphorylates AMOTL2, which is part of the Hippo pathway and promotes YAP signaling (Artinian et al., 2015). Of note, mTORC1/p70s6k and mTORC2/Akt are not separated from each other; in contrast, they jointly control the dendritic arbor morphology of the cerebellum, which is mainly that mTORC1/p70s6k drives mTORC2/Akt to work (Urbanska et al., 2012) and mTORC2/Akt can also influence mTORC1/p70s6k, whereas the detailed interaction between mTORC1/p70s6k and mTORC2/Akt needs to be further explored.

### The Role of YAP/TAZ in Angiogenesis With the Vascular Endothelial Growth Factor Pathway of Endothelial Cells

The formation of vascular networks in embryos requires angiogenesis, including sprouting angiogenesis and intussusception angiogenesis (Azad et al., 2019). Of note, the YAP expression is dynamic and widespread throughout the whole development of blood vessels. In the early stages of embryos, YAP/TAZ is predominantly located in the nucleus, whereas it is located in the cytoplasm mostly at the later period. During angiogenesis development, YAP/TAZ activity is controlled by vascular endothelial growth factor (VEGF). vascular endothelial growth factor is involved in angiogenesis, microvascular permeability, and mitosis and survival of endothelial cells (Azad et al., 2019), which may be positively correlated with PI3K and MAPK activities. vascular endothelial growth factor receptor activated by blinding to the ligand can trigger PI3K and MAPK signaling (Figure 2), which have been identified as the major inducible genes of YAP/TAZ activation. Subsequently, a large number of transcription factors, such as CYR61 and CTGF, can modulate the shape and behavior of endothelial cells associated with YAP/TAZ (Azad et al., 2019). The absence of YAP/TAZ causes major defects in retinal vascular development, including a 21% reduction in radial dilation, a 26% decrease in capillary density, and a 55% decrease in branch frequency. During vascular regression, YAP/TAZ is activated and promotes actin polymerization and CTGF induction (Brigstock, 2002; Jiang et al., 2004; Chaqour and Goppelt-Struebe, 2006; Nagasawa-Masuda and Terai, 2017), which indicates that YAP/TAZ may relieve or even restore vascular degeneration via upregulating the transcription level. Intriguingly, YAP/TAZ in the cytoplasm triggers proliferation and migration of vascular endothelial cells via regulating the Rho family GTPase CDC42 activity (Sakabe et al., 2017; Figure 2). Thus, YAP/TAZ in the endothelium is required for vascular growth, branching, and regularity of the vascular network.

Also, YAP interacts with interleukin 6 (IL-6)-driven signal trans-activator transcription 3 (STAT3; He et al., 2018) and enhances the expression of angiopoietin 2 (Ang-2), both of which trigger blood vessels generated, especially in the retina. In addition to the above, YAP/TAZ can also regulate non-endothelial cells during angiogenesis development (Azad et al., 2019), such as pericytes, edema, diabetic retinopathy, and embryonic lethality due to overdilation and blood loss of blood vessels (Bergers and Song, 2005).

## Role of YAP in Certain CNS Diseases

After in-depth research, Hippo-YAP, which is defined as the core target of certain central nervous system diseases (Liu et al., 2017; Mueller et al., 2018; Rojek et al., 2019), usually transfers from the cytoplasm that binds to TEAD to the nucleus. The binding method is that the end of N YAP interacts with the spherical structure of the C-terminal of TEAD (four α-helices around β-sheets; Chen et al., 2010; Tian et al., 2010). However, in certain central nervous system diseases, the cytoplasmic localization of YAP is widely distributed through interaction with certain specific proteins (14-3-3 protein). Thus, in various central nervous system diseases, the location of YAP and interaction with protein are ultimately different. Tumor proliferation is considered to be a prominent feature in glioma, and at the same time, Hippo-YAP expression is upregulated. Of note, YAP is related to the mutant p53, which enhances the proliferation and transcriptional activity of the tumor. The correlation between p53 and YAP can be mediated via the control of the WASP interaction protein (WIP; Zhao et al., 2007; Rivas et al., 2018), which is also regulated by the PI3K–Akt pathway. Additionally, neurofibromatosis 2 (NF2) inhibits YAP nuclear translocation and triggers the deletion of transcription factors, thereby increasing invasiveness and affecting the SATA5A–SOCS2–SATA3 pathway (Sen et al., 2012; Lee et al., 2016). In subarachnoid hemorrhage (SAH), YAP usually upregulates the expressions of Nrg1β1, ErbB4, Mst1, and PIK3CB and inhibits the cytoplasmic retention of NF-κB and the activity and degradation of MMP-9 tight junction proteins (Yan et al., 2017; Qu et al., 2018). In Huntington’s disease (HD), a mutant Huntington gene is observed, which manifests a repeat of the CAG sequence (Mueller et al., 2018). Due to the activation of Mst1 in HD, the phosphorylation of YAP increases, which induces mutations in the Huntington gene via excitotoxicity and calcium signal disturbances, mitochondrial dynamics changes, transcriptional interference, cytoskeletal disruption, and improper protein processing (Morrison, 2009; Mueller et al., 2018). Besides, in neurodegenerative diseases such as HD and Alzheimer’s disease (AD), ballooning cell death (BCD) and transcriptional repression-induced atypical cell death (TRIAD) both occur with the interaction between YAP and p73 (Pardo et al., 2006). In AD, the amyloid-β (Aβ) peptide aggregation has been proven to be a prominent feature of the pathological process. More significantly, the disease is positively correlated with the YAP/p73/Bax-mediated apoptosis and intracellular Aβ-induced neuron necrosis (Cosentino et al., 2009; Tanaka et al., 2020; Table 2).

**TABLE 2:** Summary of the regulation of YAP in central nervous system diseases.

CNS Diseases	Mechanism	Influence	References
Glioma	• Decreased cell contact inhibition • mtp53-induced tumor proliferation associated with WIP and YAP • CYR61/CCN1 and miR-296-3p mediate glioma via NF2 inhibition	Tumor proliferation of glioma has a positive correlation with higher expression and nuclear localization of YAP	(Zhao et al., 2007; Lee et al., 2016; Rivas et al., 2018)
Subarachnoid hemorrhage	• Increased Nrg1β1, ErbB4, YAP, and PIK3CB • Decreased MMP-9, degradation of tight junction proteins, and upregulation of Mst1 and NF-κB	ErbB4-YAP and Mst1 respond to early brain injury after SAH through BBB disruption, neuronal cell death, and white matter damage	(Yan et al., 2017; Qu et al., 2018; Qian et al., 2018)
Huntington disease	• A toxic effect causes the transcriptional dysregulation with five approaches • Decreased nuclear YAP induces BCD and TRIAD	Transcriptional alteration leads to mutant Huntington gene with activation of Mst1 and decreased nuclear localization of YAP in HD	(Mao et al., 2016; Mueller et al., 2018)
Alzheimer disease	• The YAP/p73-mediated apoptosis in symptomatic Alzheimer’s disease • The intracellular Aβ aggregation and YAP cytoplasmic sequestration induce Hippo pathway-dependent TRIAD necrosis in the early stage of Alzheimer’s disease	Neuron apoptosis and necrosis increase in AD via intracellular Aβ aggregation and YAP interaction with p73 or TEAD	(Zhang et al., 2011; Tanaka et al., 2020)

### Glioma

#### Characteristics of YAP Translocation Between Cytoplasm and Nucleus in Glioma

Yes-associated protein is a proline-rich phosphoprotein with two subtypes of splicing, namely, YAP1 and YAP2, mainly synthesized by the YAP1 gene. Due to this gene sequence, the subcellular localization of YAP1 is similar to YAP and has basic characteristics with YAP. In addition to the above characteristic, previous studies have revealed that increased nuclear localization of YAP can be observed in different types of cancer tissues (Zhao et al., 2007). More interestingly, YAP/TAZ activation can induce epithelial–mesenchymal transition (EMT), cell proliferation, and anti-apoptotic programs through binding to TEAD, thereby promoting the occurrence and development of tumors (Lei et al., 2008; Nishio et al., 2013; Piccolo et al., 2014). Besides, it is believed that the activation of YAP transport into the nucleus is closely related to the formation of gliomas. What’s more, the expression of Hippo signaling pathway molecules LATs2 and Mst2 and Merlin can cause cytoplasmic translocation of YAP, while phosphorylation of LATs2 significantly enhances the effect of YAP translocation (Zhao et al., 2007). Of note, effective regulation of YAP intercellular transport between cytoplasm and nucleus is essential for normal cellular development, controlling cell proliferation, organ size, and cancer development (Zeng and Hong, 2008; Harvey et al., 2013).

#### High Expression of YAP in Gliomas Is Related to NF2 Contact Inhibition and p53 Protein Variation

As a transcriptional coactivator, YAP is usually associated with oncogenes, and in many types of human cancers, elevated or overactivated expression of YAP has been observed (Bleeker et al., 2012). Glioma derives from glial cells in the brain, which can keep neurons in proper position and perform good functions. It is the most common type of tumor with about 30% occurrence ratio in central nervous system tumors and 80% occurrence ratio in malignant brain tumors (Lau et al., 2008; Goodenberger and Jenkins, 2012). Zhao et al. (2007) revealed that YAP overexpression caused the cells to fail to exit the cell cycle at confluence and to uninterruptedly grow to a higher density. At the same time, the dominant-positive YAP leads to cell transformation and escapes the inhibition of cell junctions that fundamentally destroys the limits of splitting and proliferation, which makes it difficult to control cell division and proliferation that triggers excessive cell growth and uncontrolled tumor progression (Liu and Wang, 2015) and even augments their capacity to invade host tissues and metastasize (Ribatti, 2017). However, YAP is inhibited by Merlin, which has been implicated in mediating cell contact inhibition (Okada et al., 2005; Zhao et al., 2007). In Salvador (Sav)-knockout cancer cell lines, inhibition of YAP function can restore contact inhibition in human cancer cell lines. Indeed, the loss of cell contact inhibition almost can be restored through blocking endogenous YAP function. Therefore, through the verification of YAP’s upregulation and downregulation, it can be concluded that the division and proliferation of glial cells are positively correlated with the expression of YAP.

**FIGURE 3:**
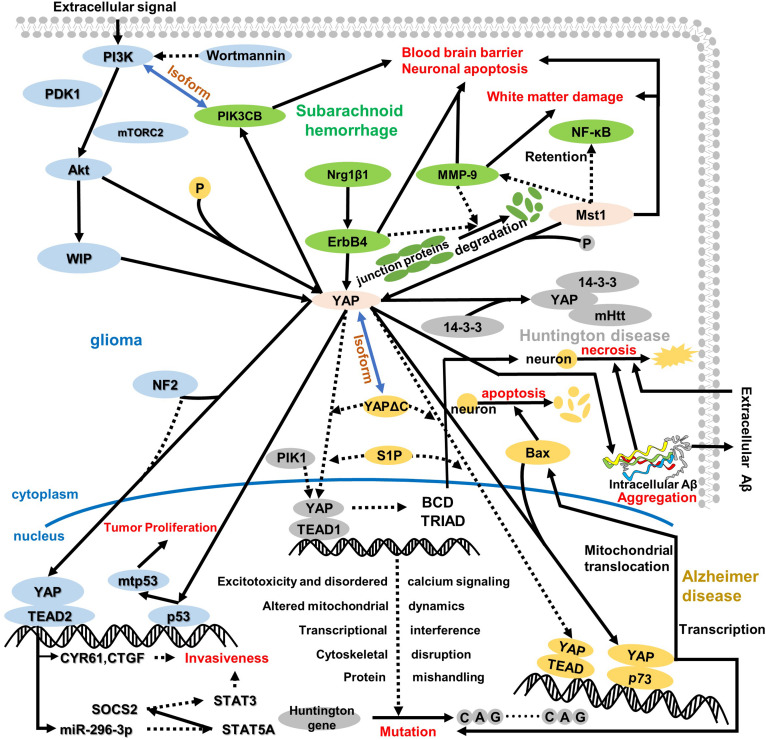
An overview of the role of YAP in the cytoplasm and nucleus regarding central nervous system disease. YAP plays a pivotal role in the whole pathologic process, which depends on its location and pathway. In glioma, YAP is regulated by WIP to induce p53 mutation in the nucleus, further resulting in tumor proliferation and attenuating contact cell inhibition. Of note, WIP expression is regulated via the P13K/Akt pathway, which is assisted by some regulatory proteins, like mTORC2 and PDK1. Meanwhile, NF2 suppresses the interaction between YAP and TEAD2, which triggers transcription target gene CYR61 and CTGF expressions and mediates miR-296-3p regulation. Both CYR61/CTGF and miR-296-3p can inhibit glioma invasiveness, whereas the invasiveness for miR-296-3p is fulfilled by decreased SATA3 and SATA5A and increased SOCS2. After subarachnoid hemorrhage, ErbB4 and Mst1 could respond to the BBB disruption, neuronal apoptosis, and white matter damage. One pathway regarding ErbB4 is upregulation of Nrg1β1, ErbB4, and PIK3CB; another pathway on Mst1 is the degradation of tight junction protein and inhibition of MMP-9 and NF-κB. Intriguingly, the isoform relationship between PI3K and PIK3CB connects glioma and subarachnoid hemorrhage. Additionally, HD is closely related to AD as a neurodegenerative disease. In HD, both decreased interaction between YAP and TEAD1 and increased YAP phosphorylation induce Huntington gene mutation. In the nucleus, the downregulation of YAP/TEAD1 stimulates CAG sequence repetition via excitotoxicity and disordered calcium signaling, altered mitochondrial dynamics, transcriptional interference, cytoskeletal disruption, and protein mishandling. Moreover, the decreased nuclear location of YAP inhibits BCD and TRIAD in cells, which stimulates neuro-necrosis. More interestingly, the mutant Huntington gene interacting with the YAP 14-3-3 complex aggravates the original pathologic symptom. In AD, YAP remarkably reduces interaction with TEAD and augments the combination with p73 that triggers Bax expression to aggravate neuron apoptosis, and YAP/p73 also aggravates the mutation in HD. Moreover, the cytoplasm retention of YAP and TRIAD/BCD mediates intracellular Aβ aggregation further, which results in neuron cell necrosis different from neuron apoptosis. Incidentally, intracellular Aβ aggregation transports outside to accumulate extracellular Aβ aggregation during the late period in AD, and extracellular Aβ aggregation can also result in neuro-necrosis with non-cell-autonomous approaches.

On the one hand, YAP can enhance the tumor proliferative transcriptional activity of mutant p53 (mtp53) protein (Di Agostino et al., 2016) through the ARF-NDM2-P53 INK4a-RB pathway, which has been defined as a significant approach of promoting oncogenic capacity. In the regulation approach between YAP and mtp53 (Rivas et al., 2018; Figure 3), the transcription of YAP/TAZ can be influenced via actin, although it is not completely dependent on actin. More obviously, WIP also mediates the YAP transcription level. Thus, WIP exerts powerful control on YAP/TAZ in glioma. Additionally, the Akt, which is considered to be a major pro-tumor factor, can regulate WIP and is then mediated by PI3K depending on phosphoinositide-dependent kinase-1 (PDK1) and mammalian target of rapamycin complex 2 (mTORC2), respectively (Yang et al., 2004; Figure 3). So Akt plays an essential role in the brand-new WIP-YAP/TAZ-mtp53 pathway (Rivas et al., 2018). However, the precise interaction mechanism between WIP and Akt is still confusing. Intriguingly, both RAC and PAK also regulate WIP besides Akt (Rivas et al., 2018).

On the other hand, the tumor suppressor NF2, a core molecule governing cell survival, motility, and invasiveness, attenuates the proliferation of glioma cells in the central nervous system (Lau et al., 2008). Of note, NF2 can induce the phosphorylation of YAP, inhibit YAP nuclear translocation, and also bind to many transmembrane receptors and intercellular proteins (Stamenkovic and Yu, 2010), like CD44, which usually promotes phosphorylation/inactivation of NF2. Meanwhile, the deletion or silencing of NF2 causes increased invasiveness of glioblastoma multiforme cells in a YAP- and TEAD2-dependent manner. When the NF2 is knocked out, YAP shifts to the nuclear rather than cytoplasm retention as the normal pathway; subsequently, the YAP target genes are highly expressed (Lee et al., 2016), such as neuronal growth regulator 1 (NEGR1), cysteine-rich angiogenic inducer 61 (CYR61/CCN1), and connective tissue growth factor (CTGF/CCN2). Among them, CYR61/CCN1 and CTGF/CCN2 have been demonstrated to be involved in the invasiveness of glioma cells, which is consistent with the research result that knockdown or silencing of CYR61/CCN1 and CTGF/CCN2 hampers the invasive phenotype (Lee et al., 2016; Figure 3). Additionally, miRNA has also been defined to control the invasiveness of glioma, especially miR-296-3p associated with increased phosphorylated STAT3 level and decreased expressions of STAT5A and SOCS2 (suppressor of cytokine signaling 2; Sen et al., 2012; Figure 3). Indeed, the regulatory functions of CYR61/CCN1, CTGF/CCN2, and miRNA are not separate; reversely, they are correlated with each other. Thus, NF2 controls the invasiveness of glioma through YAP-dependent expression of CYR61/CCN1 and miR-296-3p.

In addition to the information mentioned above, YAP also enhances the invasion and migration of glioma cells by regulating the expression and alterations of Twist and N-cadherin which could promote the transition from epithelial cells to mesenchymal cells (Goodenberger and Jenkins, 2012; Ouyang et al., 2020).

### Subarachnoid Hemorrhage

#### Major Pathological Features of Acute Period Subarachnoid Hemorrhage

SAH is a fatal disease with high mortality and poor prognosis (Connolly et al., 2012). It is identified as a subtype of hemorrhage stroke caused by the rupture of damaged blood vessels at the bottom of the brain or on the surface of the brain (van Lieshout et al., 2018). Some diseases usually are a positive correlation with SAHs, such as aneurysm and cerebrovascular malformation. More significantly, after SAH, both blood–brain barrier (BBB) disruption and neuronal apoptosis contribute to the pathogenesis of early brain injury (Yan et al., 2017). Meanwhile, after SAH, the expression levels of ErbB4 (EGFR family member v-erb-b2 avian erythroblastic leukemia viral oncogene homolog 4) were notably increased, which has been demonstrated as the pivotal mechanism of the regulation of neurite outgrowth, axonal guidance, and synaptic signaling (Shamir et al., 2012). In addition to ErbB4, Nrg1β1, YAP, and PIK3CB are all involved in early brain injury regulation within 72 h after SAH. It is worth noting that a large number of studies have indicated that ErbB4 and its downstream YAP/PIK3CB signaling pathway exert a neuroprotective effect in early brain injury after SAH. Additionally, acute white matter injury is also a crucial pathological process in early brain injury (Kummer et al., 2015; Wu et al., 2017), which is associated with the regulation of Mst1, NF-κB, MMP-9, and tight junction proteins.

#### Upregulation of YAP Is Caused by the Regulation of ErbB4 and Mst1

After SAH, the trophic factor Nrg1β1 with an epidermal growth factor (EGF)-like domain, begins to increase as early as 3 h and peaks at about 72 h, whereas endogenous ErbB4 augments at 3 h and keeps stable subsequently (Yan et al., 2017). High-dosage Nrg1β1 treatment significantly augments neurobehavioral performance, alleviates brain swelling, and attenuates brain water content, all of which contribute to BBB permeability and integrity within 24 and 72 h after SAH. The ErbB4 expression is augmented via Nrg1β1 administration; simultaneously, both YAP and PIK3CB exert the same regulatory tendency. Activation of ErbB4 via Nrg1β1 treatment alleviates the neurobehavioral deficit and attenuates the brain water content and albumin extravasation within 24 and 72 h after SAH (Qian et al., 2018). Of note, increased expressions of YAP and its downstream PIK3CB collectively protect the positive effect of ErbB4 activation about the BBB after SAH. Indeed, BBB disruption causes brain edema and brain swelling, which in turn lead to adverse outcomes, like neuronal death and neurological deficits. Thus, decreased YAP expression induces brain injury after SAH via BBB disruption. In addition to the BBB, Nrg1β1-ErbB4-YAP-PIK3CB can alleviate neurologic dysfunction and reduce neuronal cell apoptosis, which is consistent with the research result of ErbB4-knockout neuronal cell death after SAH, and the specific mechanism of action is almost similar to the mechanism mentioned above. Besides the fundamental function of the Nrg1β1-ErbB4-YAP-PIK3CB pathway (Figure 3), ErbB4 can also preserve the endothelial barrier function and attenuate the expression of inflammatory genes (Xu et al., 2005; Lok et al., 2012), which deserve to proceed with in-depth exploration.

Compared with the regulation of ErbB4 and YAP in BBB destruction and neuronal cell death, Mst1 can mediate the NF-κB/MMP-9 pathway and tight junction protein to restore white matter damage in early brain injury after SAH (Figure 3). Mst1, the upstream factor of the YAP, has been demonstrated to exert fundamental function in many central nervous system diseases, like SAH, white matter damage, and BBB disruption (Zheng et al., 2010; Lee et al., 2013; Tavares et al., 2013; Yang et al., 2016). Some experimental studies have shown that Mst1 gene knockout can improve neurobehavior and affect the expression of p65NF-κB and MMP-9, as well as tight junction proteins, such as ZO-1, occludin, and claudin-5 (Qu et al., 2018). However, the comprehensive pathway response to early brain injury is that the increased phosphorylation level of Mst1, associated negatively with NF-κB regulation in the nucleus, is negatively correlated with MMP-9 activity, which contributes to the SAH-mediated white matter injury and BBB repair via alleviating the degradation of tight junction proteins from 12 to 72 h after SAH (Pan et al., 2017). Thus, Mst1 upregulation aggravates the BBB disruption, neuronal cell death, and neurological deficits via promoting the NF-κB/MMP-9 pathway and attenuating tight junction proteins.

Overall, during SAH, the BBB, white matter damage, neuronal apoptosis, and neurobehavioral defects play a role in early brain damage. In view of physiological homeostasis, the regulation of Nrg1β1, ErbB4, YAP, and PIK3CB all increased, while the regulation of Mst1, NF-κB, MMP-9, and tight junction protein increased early brain damage. During SAH, the variation of Hippo-YAP and Mst1 reached consistency, but the effect was completely opposite. More interestingly, the start times of Nrg1β1 and Mst1 are 3 and 12 h respectively, which is worth pondering.

### Huntington’s Disease

#### Mutant Huntington Gene of Chromosome 4 Causes Classical Neurodegenerative Diseases

HD is an autosomal dominant hereditary neurodegenerative disorder characterized by progressive dyskinesia, progressive cognitive decline, and mental disorders, usually within 15–20 years of diagnosis. The major manifestation of dyskinesia is chorea associated with degeneration or hereditary defects of GABAergic interneurons in the neostriatum and striatum atrophy (Lasker et al., 1987). Meanwhile, the main clinical manifestations of cognitive impairment are the decline of memory, attention, and language function (Paulsen et al., 2013), and mental disorders are principally emotional outbursts, depression, and anxiety. More significantly, the disease is almost caused by a mutation in the Huntington gene of chromosome 4, subsequently producing mutant proteins, which continuously accumulate in the central nervous system and finally intervene in the normal function of the nerve cell (Dayalu and Albin, 2015). Previous studies have indicated that the extended CAG repeat sequence in the Huntington gene, averaging 17–20 CAG repeats (Kremer et al., 1994), leads to an over-long polyglutamine extension near the N-terminus of the protein. Interestingly, the occurrence and development of HD are due to the CAG gene being repeated at least 40 times, which has been proven (Mueller et al., 2018). Therefore, mutations in the Huntington gene on chromosome 4 can cause CAG duplication, which can easily lead to HD.

#### Activation of Mst1 and the Decrease of YAP Nuclear Localization Induce the Transcriptional Change of the HD Gene

Many research results have demonstrated that both activation of Mst1 and the decline of nuclear YAP expression lead to transcriptional dysregulation with the occurrence of HD (Mueller et al., 2018). Thus, the Mst1 activation and the YAP phosphorylation suppress the normal transcriptional function and induce the mutation of the Huntington gene, both of which contribute to the occurrence of HD. Due to cytoplasm retention of YAP, YAP frequently binds to the chaperone 14-3-3 regarded as an abundant protein in the brain before degradation (Morrison, 2009). Correspondingly, decreased YAP nuclear localization may translate to alterations in gene expression via reducing YAP/TEAD interactions. During HD, transcriptional dysregulation is a pivotal pathogenic mechanism (Cha, 2007; Glajch and Sadri-Vakili, 2015), just like the mutant Huntington gene. Of note, the cortex and striatum have been observed to have increased phosphorylated Mst1 and YAP levels, the combination of YAP and TEAD in the nucleus is decreased when transcriptional dysregulation occurs, and the alteration of Mst1 phosphorylation and YAP nuclear localization is almost basically consistent via LATs1/2-mediated activation (Mueller et al., 2018). In exactly the above alteration, the transcription disorder induces the mutation of the Huntington gene, especially CAG sequence repetition (Figure 3). More interestingly, with the disruption of the YAP/TEAD interaction, the transcriptional dysregulation is accompanied by a toxic effect during Huntington gene mutation, mainly the following five core mechanisms:

(1) *Transcriptional interference*. The mutant Huntington gene enters the nucleus and directly interferes with gene transcription, thereby affecting neurotransmitter receptors and ion channels (Hodges et al., 2006), as well as BDNF (Zuccato et al., 2001).
(2) *Cytoskeletal disruption*. The mutant Huntington gene affects the microtubule system, leading to failure of vesicle transport (Caviston and Holzbaur, 2009).
(3) *Protein mishandling*. The mutant Huntington gene may overwhelm the neuron’s ability to tag and clear degraded and misfolded proteins via the ubiquitin–proteasome (Wang et al., 2008) and autophagy–lysosome (Wong and Holzbaur, 2014) pathways.
(4) *Altered mitochondrial dynamics*. The mutant Huntington gene alters many mitochondria-associated messenger RNA levels (Shirendeb et al., 2011); reduces transcription of PGC-α, which itself is a pivotal transcriptional regulator to activate a series of important roles in mitochondrial structure and function (Weydt et al., 2006); and leads to a decline in ATP levels and oxidative stress (Mochel et al., 2012), which all cause brain metabolic defects and increased cerebrospinal fluid lactate levels (Koroshetz et al., 1997).
(5) *Excitotoxicity and disordered calcium signaling*. The mutant Huntington gene directly sensitizes NMDA receptors via the NR2B subunit, which results in excitotoxicity (Zeron et al., 2001). Meanwhile, NMDA results in excessive calcium influx by interacting with type 1 inositol triphosphate receptors, which all exert huge pressure on neurons and ultimately result in cell abnormity and apoptosis (Zeron et al., 2002).

Given the above mechanism regarding the mutant Huntington gene, a slightly integrated pathway can be summarized in which both Mst1 activation and YAP phosphorylation attenuate the combination of YAP and TEAD via reducing the nuclear translocation of YAP, which all induce transcriptional interference, cytoskeletal disruption, protein mishandling, altered mitochondrial dynamics, and excitotoxicity and disordered calcium signaling to cause the Huntington gene mutation, like the CAG repeat that easily occurs in HD.

In addition to the above mechanism, decreased nuclear YAP induces TRIAD (Pardo et al., 2006), which is similar to mutant-Huntington-mediated neuronal death termed BCD rather than the p73/YAP-induced apoptosis. Intriguingly, both YAP and YAPdeltaC can recover BCD and TRIAD via binding to TEAD1 that is validated as a target transcription in BCD (Mao et al., 2016). BCD has proven to be a type of necrotic cell death, similar to TRIAD, which expands the ER and destroys asymmetric cell bodies. Therefore, YAP nuclear translocation attenuates BCD in mutant Huntington by necessarily binding to TEAD1. Additionally, PIK1 has been certified to switch the partner of YAP from TEAD1 to p73, which forms the balance of TEAD/YAP-dependent necrosis and p73/YAP-dependent apoptosis (Mao et al., 2016; Figure 3). However, SIP can promote the combination of TEAD1 and YAP. Unfortunately, this theory has not yet been applied to HD maturely, but interventions for TRIAD and BCD may lead to the discovery of new therapeutic targets for HD.

### Alzheimer’s Disease

#### Classical Features of Alzheimer’s Disease

Alzheimer’s disease is a common neurodegenerative disease in the central nervous system with progressive cognitive disorder and neurobehavioral disruption. Its pathological features are amyloid plaques, neurofibrillary tangles, and neuronal loss (Ikonomovic et al., 2008). Indeed, the deposition of Aβ peptide in the brain has been demonstrated as a pathological hallmark of AD, and it has also been validated to result in neurotoxicity and cell apoptosis *in vivo* and *in vitro* (Cancino et al., 2008; Lauren et al., 2009). Intriguingly, multiple types of previous experimental studies indicated that neuronal cell apoptosis was implicated in the pathogenesis of AD (Yuan and Yankner, 2000; Gupta et al., 2006). In addition to the Aβ peptide deposition, the pathogenesis of AD is closely associated with tau protein neurofibrillary tangles (Xia et al., 2017), neuroinflammatory mechanism theory (Kamat et al., 2016; Trovato Salinaro et al., 2018), and neurovascular damage theory and oxidative stress (Zhao and Zhao, 2013). In the following section, the relationship between pathological hallmarks of AD and YAP could be emphatically elaborated.

#### p73-Mediated Apoptosis and Intracellular Aβ-Induced Neuron Necrosis

During symptomatic AD, YAP is implicated in the deposition of the Aβ peptide via an apoptotic medium, which differs from the necrosis in AD. To be precise, apoptosis plays a vital role in the body’s physiological survival, such as cell renewal and homeostasis, but it can also lead to diseases such as AD (Gupta et al., 2006; Cancino et al., 2008; Zhao et al., 2008c). As a key factor in inhibiting the apoptosis pathway, the nuclear YAP expression level of neurons during AD decreased significantly, and the cytoplasmic retention of YAP was severely phosphorylated. The retention of YAP in the cytoplasm usually reduces the interaction with TEAD1. If YAP is transferred from the cytoplasm to the nucleus, it can be combined with p73 to promote apoptosis and necrosis (Basu et al., 2003; Strano et al., 2005; Hoshino et al., 2006; Levy et al., 2008), rather than promoting cell proliferation, differentiation, and survival through TEAD interaction (Saucedo and Edgar, 2007; Zhao et al., 2008a, 2010b). Besides binding to p73, YAP maintains the protein stabilization to augment p73-mediated apoptosis. After the interaction between YAP and p73 in the nucleus, the complex stimulates pro-apoptotic gene Bax mitochondrial translocation rather than direct transcription (Zhang et al., 2011). Interestingly, the gene Bax is positively correlated with p73-induced mitochondrial translocation and neuron apoptosis (Figure 3).

However, in the early stage of AD, including preclinical AD, mild cognitive impairment, or ultra-early stage of AD, YAP deprivation mediates Hippo pathway-dependent necrosis via intracellular Aβ, which is similar to that induced by YAP cytoplasmic retention in mutant Huntington gene (Mao et al., 2016) and differs from the apoptosis belonging to type II cell death. Of note, during AD, numbers of intracellular Aβ and TEAD-YAP transcription are closely related to ER ballooning and the Hippo pathway-dependent TRIAD. Before ER ballooning, intracellular Aβs were increased, and TEAD-YAP transcriptional activity was reduced. Thus, intracellular Aβ aggregation is inversely correlated in the transcription of TEAD and YAP (Figure 3). In addition to the above in AD, the Hippo pathway-dependent TRIAD necrosis has been validated as a pivotal role in AD pathology, and the necrosis ultimately depends on the correlation of intracellular Aβ and YAP (Tanaka et al., 2020). Therefore, the approaches between YAP and necrosis could be elucidated as follows (Strano et al., 2005; Levy et al., 2008):

(1) Due to intracellular Aβ-mediated necrosis, vast quantities of cerebral neurons are remarkably decreased through the YAP retention during a cell-autonomous degradative process.
(2) Secondary cell damage is implicated in necrotic neurons via release of alarmins to expand bystander neuron degeneration during a non-cell-autonomous process.
(3) Extracellular Aβ, which is aggregated via the medium of intracellular Aβ, aggravates degeneration by another non-cell-autonomous approach.

Therefore, the Hippo pathway-dependent TRIAD necrosis and intracellular Aβ aggregation are induced by YAP deprivation, occurring most actively in the early stages of AD before extracellular Aβ aggregation.

More intriguingly, both S1P and YAPdeltaC have been validated as a candidate therapeutic strategy in human AD. Slices of results have revealed that S1P and YAPdeltaC reduced the extracellular Aβ burden and increased nuclear YAP/YAPdeltaC, which decreases ER instability, necrosis, and cognitive disorder. Thus, the repression of TEAD-YAP/YAPdeltaC-induced transcription could be rescued via S1P and YAPdeltaC under YAP sequestration into intracellular Aβ aggregates, which was not affected by the treatments. Additionally, due to Akt activation, the wortmannin, an inhibitor of PI3K upstream of Akt, could remarkably abolish Akt-induced protection from apoptosis Aβ_25__–__35_-treated cells (Figure 3).

Collectively, in AD, neuron cell death occurs throughout the whole degradative process, including apoptosis and necrosis, both of which are correlated with YAP and intracellular Aβ. During the apoptosis of AD, nuclear YAP binds to p73 which induces cell apoptosis to express the transcription gene Bax, which has been certified as a pivotal pro-apoptotic target (Mao et al., 2016). Additionally, in the early stage of AD, both intracellular Aβ aggregation and YAP sequestration are positively correlated with the Hippo pathway-dependent TRIAD neuron necrosis through about three expansive approaches (Tanaka et al., 2020). With the in-depth exploration, slices of experimental results have revealed that S1P, YAPdeltaC, and wortmannin may suppress Aβ aggregation and cell death via increasing YAP/YAPdeltaC nuclear translocation.

## Conclusion

Collectively, YAP plays a pivotal role in the healthy and pathologic central nervous system. The role of YAP in the central nervous system is diverse due to its location and pathway with other proteins or genes, which are all based on cell proliferation, apoptosis, cell contact inhibition, and organ volume control. Thus, portions of increased YAP expression can reduce cell death, thereby ameliorating the original pathologic symptoms, like neuroinflammation, whereas parts of decreased expression aggravate adverse elements. In a healthy central nervous system, we unravel some correlation between healthy cells and YAP, such as NPC, BV-2 neuroglia cells, Purkinje cells, and endothelial cells. The specific function includes a balance between proliferation and apoptosis, the response to neuroinflammation with Sirt3 and JNK pathways, the smooth signal transmission with mTORC1/p70s6k and mTORC2/Akt regulation, and the formation of vascular networks with CTGF and CYR61. In addition to the healthy system, glioma, SAH, HD, and AD are all elucidated as part of CNS diseases. In glioma, the YAP expression is upregulated and implicated in the mutant p53, which enhances the tumor proliferative transcriptional activity. Besides, YAP nuclear translocation is inhibited through NF2 to aggravate tumor invasiveness related to miR-296-3p and CTGF/CYR61. In SAH, the YAP is positively correlated with the regulation of Nrg1β1, ErbB4, Mst1, and PIK3CB, whereas inversely with NF-κB cytoplasmic retention, MMP-9 activity, and degradation of tight junction proteins. Additionally, Huntington gene mutation, observed in HD, is induced via increased YAP phosphorylation and TRIAD/BCD-mediated cell death. Ultimately, the Aβ peptide aggregation has been validated as a remarkable characteristic of the AD pathology process, which is closely associated with the intracellular Aβ-induced neuron necrosis and YAP/p73-mediated apoptosis.

## Author Contributions

All authors listed have made a substantial, direct and intellectual contribution to the work, and approved it for publication.

## Conflict of Interest

The authors declare that the research was conducted in the absence of any commercial or financial relationships that could be construed as a potential conflict of interest.
